# Recent advances in drug delivery systems for enhancing drug penetration into tumors

**DOI:** 10.1080/10717544.2020.1831106

**Published:** 2020-10-26

**Authors:** Bin He, Xin Sui, Bing Yu, Song Wang, Youqing Shen, Hailin Cong

**Affiliations:** a Institute of Biomedical Materials and Engineering, College of Chemistry and Chemical Engineering, College of Materials Science and Engineering, Affiliated Hospital of Qingdao University, Qingdao University, Qingdao, China; b Key Laboratory of Bio-Fibers and Eco-Textiles, College of Chemistry and Chemical Engineering, Qingdao University, Qingdao, China; c Center for Bionanoengineering and Key Laboratory of Biomass Chemical Engineering of Ministry of Education, College of Chemical and Biological Engineering, Zhejiang University, Hangzhou, China

**Keywords:** Tumor penetration, drug delivery systems, microenvironment, nano-drugs, endocytosis

## Abstract

The emergence of nanomaterials for drug delivery provides the opportunity to avoid the side effects of systemic drug administration and injury caused by the removal of tumors, delivering great promise for future cancer treatments. However, the efficacy of current nano drugs is not significantly better than that of the original drug treatments. The important reason is that nano drugs enter the tumor vasculature, remaining close to the blood vessels and unable to enter the tumor tissue or tumor cells to complete the drug delivery process. The low efficiency of drug penetration into tumors has become a bottleneck restricting the development of nano-drugs. Herein, we present a systematic overview of recent advances on the design of nano-drug carriers in drug delivery systems for enhancing drug penetration into tumors. The review is organized into four sections: The drug penetration process in tumor tissue includes paracellular and transcellular transport, which is summarized first. Strategies that promote tumor penetration are then introduced, including methods of remodeling the tumor microenvironment, charge inversion, dimensional change, and surface modification of ligands which promote tissue penetration. Conclusion and the prospects for the future development of drug penetration are finally briefly illustrated. The review is intended to provide thoughts for effective treatment of cancer by summarizing strategies for promoting the endocytosis of nano drugs into tumor cells.

## 1. Introduction

The principal clinical treatment modalities for cancer currently involve surgical resection, radiotherapy, chemotherapy, and immunotherapy (Rampling et al., 2004; Sun et al., 2014). Early and mid-term cancers can be treated by surgical resection, but this is not applicable in terminal cancer. Radiotherapy can be used for the treatment of advanced cancer (DeSantis et al., 2014), but it is not selective. It kills both cancer and normal cells, resulting in multiple side effects (Earlam & Cunha-Melo, 1980). Tumor immunotherapy relies mainly on the regulation or activation of the host’s immune system to suppress or kill tumors, which has the advantages of low toxicity and high efficiency (Green et al., 2001; Sharma et al., 2011). However, due to an immunosuppressive microenvironment in solid tumors, some immune cells or cytokines administered into the vascular system cannot successfully reach the tumor (Chen et al., 2015; Sun et al., 2020). Although immunotherapy is effective in tumors of the blood, the effect is not obvious when applied to solid tumors (Jiang et al., 2017). Chemotherapy is a systemic method of treatment (Pathak et al., 2020). Chemotherapeutic drugs are spread through blood circulation to most tissues and organs of the patient, so it is the principal method of treatment for blood and metastasized advanced tumors (Wu & Chang, 2010). Chemotherapy using small molecule drugs, such as doxorubicin (Cohen et al., 2012), camptothecin (Liu et al., 2008), paclitaxel (Von Hoff et al., 2011), or cisplatin (Siddik, 2003) are the most commonly-used anti-tumor treatments (Sherman-Baust et al., 2011). All have issues of water solubility (Sanches & Ferreira, 2019), structural stability, pharmacokinetic distribution (Rosso et al., 2009) and biochemistry (Carvalho et al., 2014; Gunasekaran et al., 2014), resulting in low effective drug concentrations in tumor tissues. The emergence of nanocarrier materials solves these problems and avoids the side effects of drugs, representing considerable promise for future cancer treatments (Meng et al., 2019).

Compared with traditional chemotherapy, nano-drug delivery systems can safely and effectively deliver therapeutic drugs to target cells, thereby avoiding undue effects on healthy cells and adverse toxicity in patients (Cong et al., 2020). However, in clinical applications, nano-drug has only been shown to reduce the toxicity and side effects of drugs and does not significantly improve efficacy compared with traditional methods of drug administration (Matsumura & Maeda, 1986). The principal reason is that the drugs do not infiltrate well into the cells and tissues of tumors located far from blood vessels (Durymanov et al., 2015), especially in hypoxic regions (Movsas et al., 1999; Bache et al., 2008), and do not easily undergo intracellular drug entry or release, resulting in unsatisfactory efficacy. Chemotherapeutic drugs generally provide anti-tumor effects through the inhibition of cell proliferation or DNA replication and can only cause this effect at a particular concentration (Sui et al., 2011). In regions of tumor tissue, especially in deep parts far from blood vessels, the drug concentration is almost zero due to the low osmotic capacity of nano-drugs, so efficacy cannot be achieved. The low permeability of solid tumors has become a bottleneck restricting the development of nano-drugs (Maeda et al., 2000). Therefore, a deeper understanding of the reasons for the low levels of nano-drugs penetration and promotion of nano-drug endocytosis into cells is key to solving this problem (Nakamura et al., 2016).

The reasons for poor nano-drug penetration into tumor tissues relate to the size of the nano-drug and pathological features of the tumor tissue. Positive charges on their surface can promote integrated endocytosis of nano-drug systems into cells, which promote their penetration into tumor tissue (Gratton et al., 2008; Chen et al., 2016; Pang et al., 2016). However, this increases recognition by the reticuloendothelial system in the blood circulation, accelerating the rate of clearance and reducing its accumulation in tumor tissue (Chen et al., 2017). In addition, a large size (∼100 nm) can result in a longer persistence of circulation of nano-drug in the blood, which is not consistent with tissue penetration. Nano-drugs that are smaller (<20 nm) more effectively penetrate into tumor tissues, but this leads them to be more rapidly cleared from the blood. For the delivery of nano-drugs, carriers are required to overcome different biological barriers, some even requiring contradictory strategies. Therefore, achieving greater drug delivery efficiency requires dynamic strategies designed to regulate the properties of nano-drugs. Herein, we present a systematic overview of the strategies that promote drug penetration and discuss the design of nano-drugs. The drug penetration process in tumor tissue involving paracellular and transcellular transport (Bugno et al., 2016) is summarized first, after which strategies for the promotion of tumor penetration are introduced, including remodeling of the tumor microenvironment, charge inversion, dimensional change and surface modification of ligands which promotes tissue penetration. Finally, the prospects for the future development of drug penetration are briefly illustrated.

## 2. Drug delivery process

**Figure 1.**
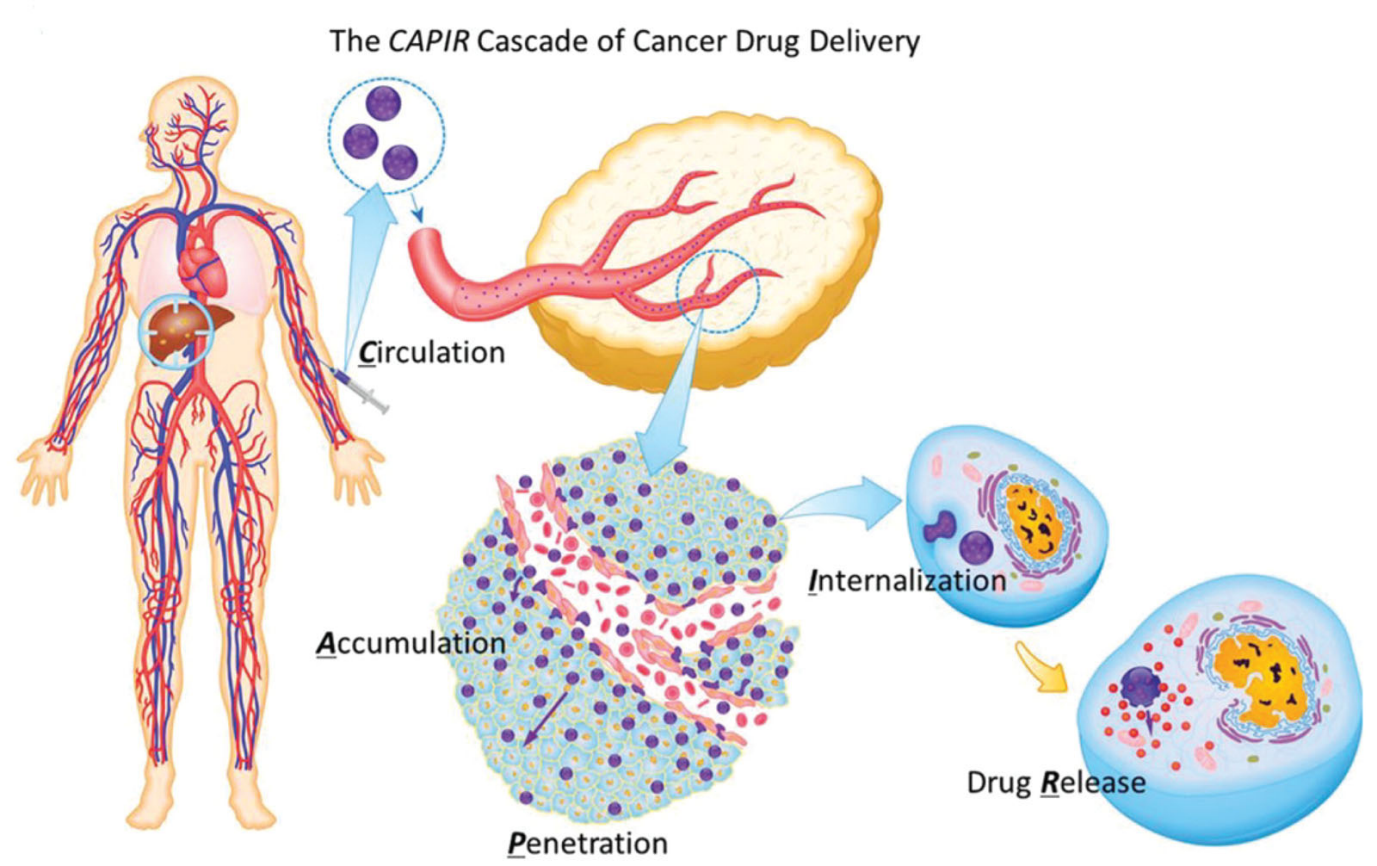
CAPIR five-step cascade process (Sun et al., 2014).

Nanocarriers have to overcome a series of biological barriers in order to deliver drugs into solid tumors (Blanco et al., 2015). For example, after intravenous injection of a cancer nano-drug system, it resides in the blood circulation (C) for transportation throughout the body (Maeda et al., 2013). The EPR effect (Padera et al., 2002) results in nanoparticle accumulation near the tumor (A) after which the nano-drug penetrates deep into the tumor tissue (P). The cancer cells internalize the drug (I) causing the nanocarriers to release (R) the cancer nano-drugs. These five steps are referred to as the ‘CAPIR’ cascade (Figure 1) (Sun et al., 2014). Only when the ‘CAPIR’ process is successfully implemented can a therapeutic effect be guaranteed (Zhong et al., 2020). In this process, the steps P and I are the most difficult to achieve and represent a major obstacle that restricts cancer treatment.

In terms of this 5 step cascade, researchers have achieved considerable success in long cycling (C) (Klibanov et al., 1990; Hu et al., 2011), tumor enrichment (A) (Wu et al., 2014; Song et al., 2015), enhancement of intracellular endocytosis (I) (Mizuhara et al., 2015; Deng et al., 2016), and intracellular release (R) (Yu et al., 2015; Ahn et al., 2018), *etc*., but the penetration of nanomaterials into tumor tissues (P) remains the bottleneck in nanomaterial delivery (Kim et al., 2017), preventing intracellular entry (I) and release (R) into tumor cells far from blood vessels, especially in hypoxic regions, resulting in unsatisfactory efficacy, an important reason for the failure of many recently developed nano-drugs in clinical trials. For example, DOXIL (Barenholz, 2012), a nano-drug consisting of doxorubicin hydrochloride in liposomes, was able to circulate steadily in the blood for several days, with a significantly higher concentration of DOXIL at the tumor site than that of a control group injected with the small molecule adriamycin. However, the final therapeutic effect was similar in both groups, failing to significantly improve drug efficacy. The study demonstrated that a large quantity of DOXIL was concentrated around the tumor blood vessels and did not spread further to tumor cells far from the blood vessels, thus failing to undergo intracellular entry and finally drug release (Kohli et al., 2014).

The reasons for difficulties in achieving nano-drug penetration into tumor tissues can be explained by two factors: 1) the size of the nano-drug itself: nano-drugs range from a few nanometers to more than 100 nanometers. The diffusion rate is inversely proportional to its size, so the diffusion capacity of large nano-drugs is considerably smaller than that of small molecules. 2) Pathological features of tumor tissue: tumor tissue has unique physiological and pathological features such as very dense stromal tissue, high cell density, high interstitial pressure and the lack of a capillary network caused by unrestricted cell proliferation, making it very difficult for the nanomaterials to penetrate and spread within the tissue (Scott et al., 2009). Additionally, many tumors are distant from the blood vessel network and become hypoxic. Tumor cells in hypoxic regions have strong drug resistance and metastatic capability, and even small molecule drugs are found in low concentrations in such regions.

**Figure 2.**
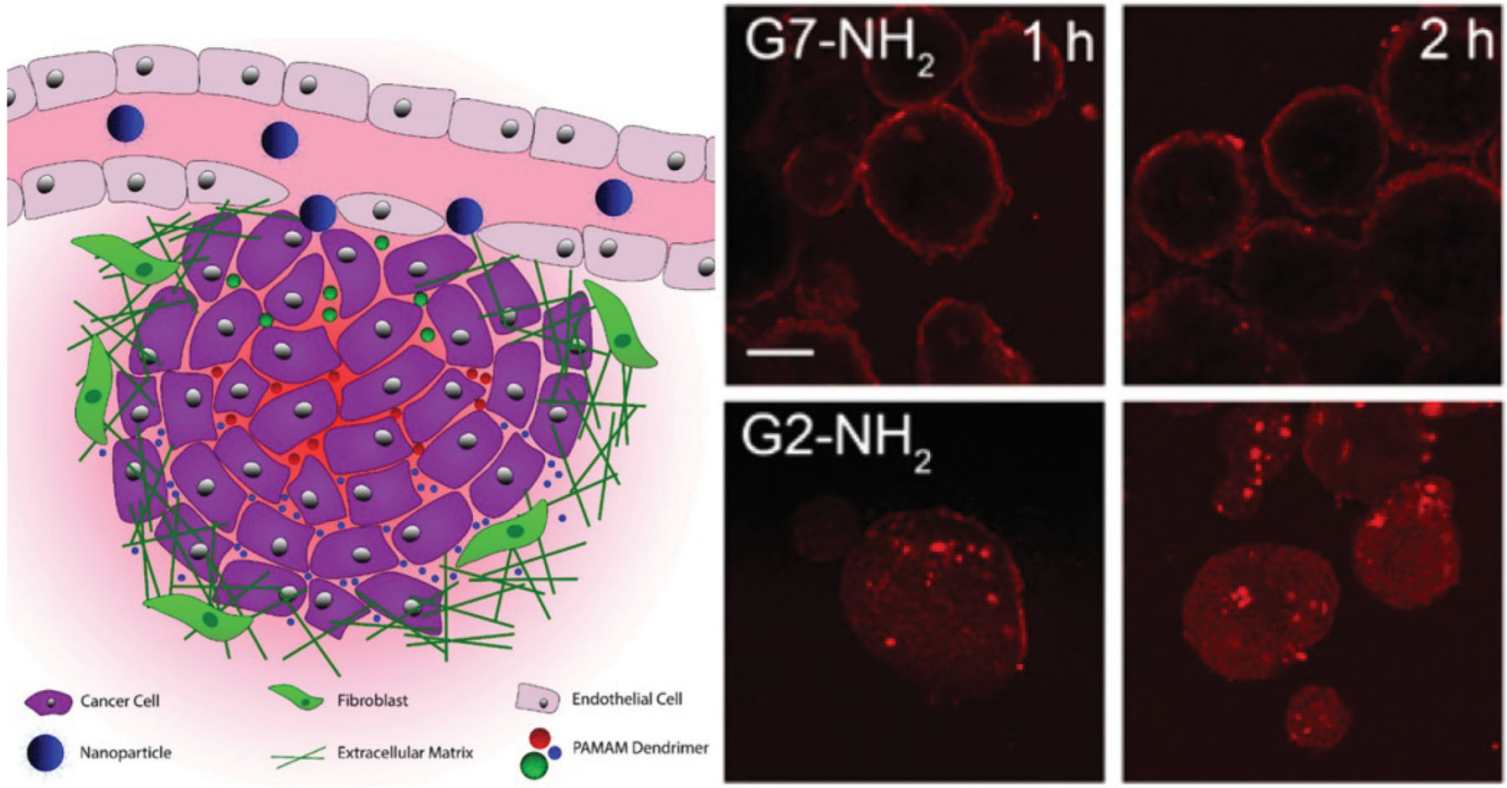
Penetration behavior of the PAMAM dendrimers using paracellular (G2) or transcellular transport (G7) (Bugno et al., 2016).

In order to achieve better penetration of nano-drugs into tumor tissues, it is important to understand how penetration occurs. There are a variety of penetration modes for different nano-drug delivery systems into tumor tissue, being described as either paracellular or transcellular transport. For example, Figure 2 demonstrates the mechanism of penetration by PAMAM dendrimers using paracellular (G2) or transcellular (G7) transport (Bugno et al., 2016). The nature of the nanocarrier determines the mode of penetration. The size of the carrier, its surface charge, the manner the drug enters cells, and the modified targeting polypeptide all affect penetration behavior in tumor tissues.

### 2.1. Paracellular transport

**Figure 3.**
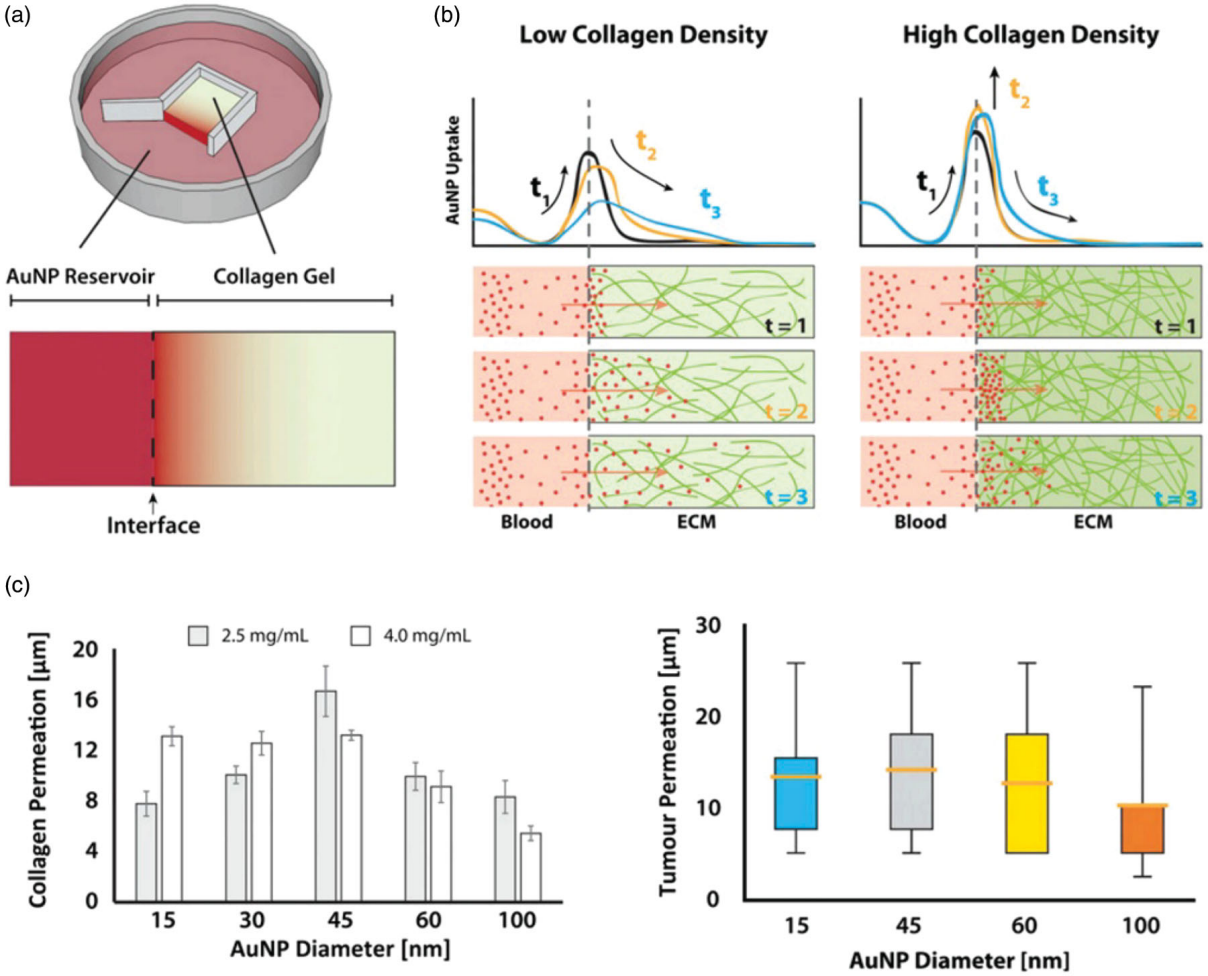
(a) Schematic diagram depicting in vitro collagen model of AuNP transport through tumor ECM and illustration of the observed AuNP (red) infiltration process in collagen hydrogels (green). (b) The permeation of AuNPs within the collagen hydrogels after 900 mins exposure. (c) Whisker plots depicting the cumulative penetration of AuNP from blood vessels into tumor tissues (Sykes et al., 2016).

Bugno et al. (2016) prepared a series of PAMAM dendrimers varying in size: G2-NH_2_, G4-NH_2_, and G7-NH_2_, and compared their permeability in an extracellular matrix (ECM) model and a 3 D multicellular tumor spheroid (MCTS) model. The results demonstrated that the smaller PAMAM dendrimer (G2-NH_2_) not only diffused faster in the ECM model but was also more effective in penetrating the MCTS core compared with the larger molecule (G7-NH_2_). The higher generation PAMAM (G7-NH_2_) was not found in significant quantities inside the tumors as large molecules only penetrated cells through cell-to-cell transmission, moving through internalization. In addition, Cabral et al. (2011) compared the duration of circulation and permeability of polymer micelles with their diameter (30, 50, 70, and 100 nm) in tumor environments of low and high osmolality. All polymer micelles were able to penetrate tumors of high permeability in mice, but only 30 nm micelles could penetrate low permeability tumors and exhibit an anti-tumor effect. Therefore, only drug delivery systems having a small particle size are able to infiltrate tumors, by paracellular transport. Although a nano-drug permeation model of tumor interstitium (ECM) is difficult to construct, it was simulated by Sykes et al. (2016) using collagen of different densities to study the penetration of varying sizes of gold nanoparticle (AuNP) (Figure 3(a)). They found that the process of ECM stromal infiltration by AuNPs could be considered as two steps: (1) Rapid enrichment on the substrate surface; (2) Gradual permeation of the AuNPs into the matrix from concentrated regions. The permeation of AuNPs into collagen hydrogels after 900 min postexposure is displayed in Figure 3(b), with increasing collagen density weakening AuNP nanoparticle permeability. Whisker plots confirm cumulative AuNP penetration from blood vessels into tumor tissues (Figure 3(c)).

### 2.2. Transcellular transport

**Figure 4.**
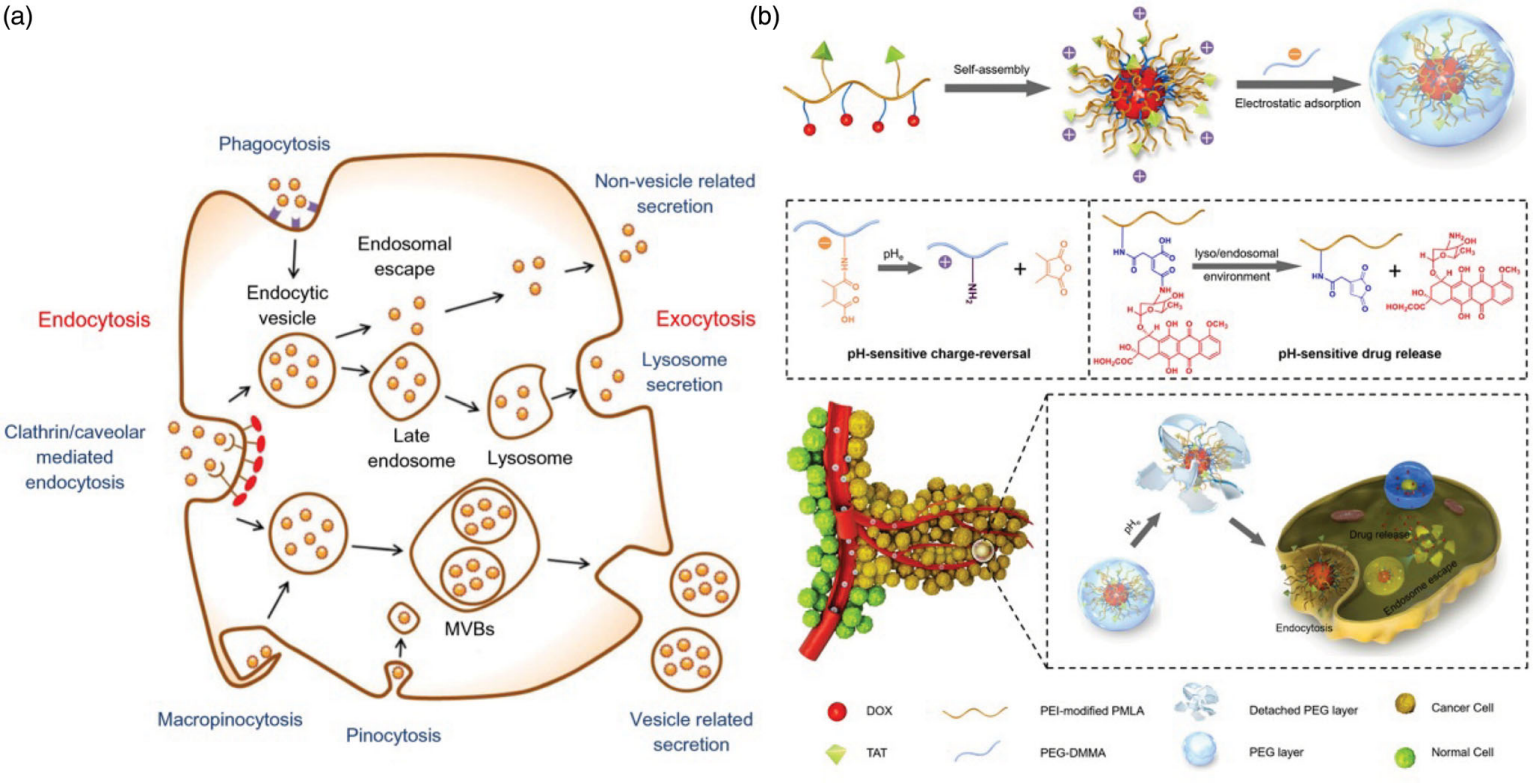
The four pathways of cellular uptake of nanoparticles: clathrin-/caveolar-mediated endocytosis, phagocytosis, macropinocytosis, and pinocytosis. The three types of exocytosis include lysosome secretion, vesicle-related secretion, and non-vesicle-related secretion (Oh & Park, 2014). (b) Preparation of the pH-sensitive drug delivery system PMLA-PEI-DOX-TAT@PEG-DMMA, and schematic illustration of the dual pH-sensitive DOX-loaded nano-complex with charge-conversion function for effective tumor-targeted drug delivery and enhanced cellular uptake (Zhou et al., 2017a).

As described earlier, where nanoparticles reach tumors by the blood circulation, smaller nanoparticles can penetrate into their interior through the stroma, while larger-sized nanoparticles are not able to penetrate as deep through this pathway, more likely entering the interior of cells by endocytosis. Not all nanoparticles that enter cells are digested. After nano-drugs are integrated into tumor cells, many nano-particles will be discharged via a variety of pathways and taken up by adjacent tumor cells allowing delivery of the nano-drugs to multiple neighboring cells. Hence, transcellular transportation includes the endocytosis and exocytosis of nanoparticles. There are four principal pathways that cells use to take up nanoparticles: clathrin-/caveolar-mediated endocytosis, phagocytosis, macropinocytosis, and pinocytosis (Xiao et al., 2019). Nanoparticles taken into cells form endocytic vesicles. Some endocytic vesicles aggregate to form multivesicular bodies (MVBs) and are then exocytosed to the cell exterior. Some nanoparticles are internalized by the cell and enter endosomes, entering early lysosomes, those that escape taken up by the Golgi apparatus or endoplasmic reticulum, finally being secreted out through the Golgi apparatus (Yanes et al., 2013; Ding et al., 2017). The remaining nanoparticles in early lysosomes are finally discharged from cells through late lysozymes in vitro (Figure 4(a)) (Oh & Park, 2014).

A requirement of the delivery of nano-drugs into cancer cells is that the maximum possible quantity is transferred. Nanoparticles with positively-charged surfaces interact with negatively charged cell membranes, effective in promoting cell internalization (Mintzer & Simanek, 2009; Aoshima et al., 2013). Zhou et al. (2017a) prepared a dual-pH sensitive charge-reversal nano-complex, in which polyethylenimine (PEI) was used to modify poly (β-L-malic acid) (PLMA) to prepare PMLA-PEI (Lee et al., 2006; Huang et al., 2012), causing the entire system to be positively charged. pH-sensitive cis-aconitic anhydride-modified doxorubicin (DOX-CA) was then attached to PMLA-PEI (PMLA-PEI-DOX), to which the cell-penetrating peptide TAT (Lange et al., 2007) was introduced to further improve its tumor-penetrating capability. Finally, it was PEGylated (Gratton et al., 2008; Knop et al., 2010; Lankveld et al., 2011) into a drug carrier with pH responsiveness and good cell penetration capability (PMLA-PEI-DOX-TAT@ PEG-DMMA) (Figure 4(b)). When the drug-loaded system arrives at the tumor through blood circulation, the nanoparticles are taken up into cells by becoming positively charged. Following ingestion, the drug-loaded system releases the nano-drugs, some becoming secreted into the tumor stroma by lysosomes, and other cells inside the tumor further ingesting the drugs, achieving transcellular drug delivery. Ju et al. (2014) constructed a pH-responsive nano hydrogel, NLSC-NG, designed to aggregate at the sites of tumors due to the weak acidic tumor microenvironment and become ingested. Some drugs entering cells also become secreted, to be ingested by deeper within the tumor, representing transcellular delivery of the nano-drug.

**Figure 5.**
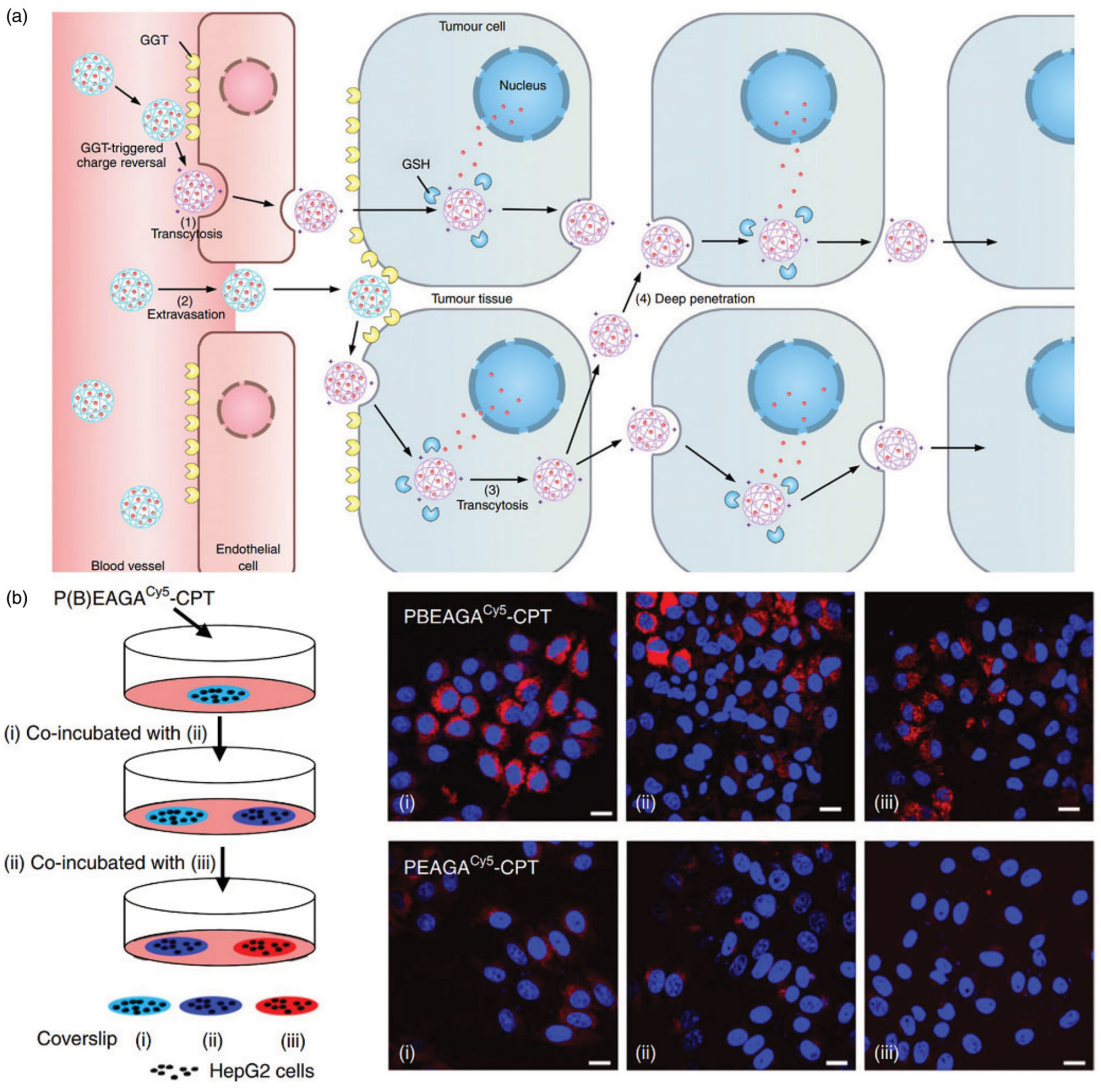
(a) Illustration of cationization-initiated transcytosis mediated active tumor penetration for transendothelial and transcellular transport of a nano-drug. (b) Intracellular transfer of PBEAGA^Cy5^-CPT visualized by confocal microscopy (Zhou et al., 2019).

Zhou et al. (2019) designed and synthesized a γ-glutamyl transpeptidase (GGT)-responsive charge-reversal polymer (PGABEA-GGT), which was negatively-charged in blood, the attached PEG exhibiting an outstanding half-life within the circulation. The high expression of GGT around the blood vessels following the entry of the polymer into the tumor catalyzes the charge reversal, allowing the polymer drug-loaded system to easily penetrate into cells leading to intracellular drug release. High levels of tumor suppression have been verified using animal models. The mechanism of the high levels of permeability of PGABEA-GGT in tumor tissue has been studied, finding that PGABEA-CPT rapidly penetrated tissues via cell-to-cell transmission through an endocytosis-exosmosis pathway mediated by the cell membrane (Syvanen et al., 2017). It was proposed that in addition to enhancing passive accumulation through osmosis and maintenance effects (Shi et al., 2017) (Figure 5(a) (2)), endothelial cell endocytosis was also able to actively transport nano-drugs to tumor tissues through capillary walls (Figure 5(a) (1)). Furthermore, this ATP-dependent mechanism also bypassed the passive diffusion barrier described above, allowing nano-drugs to actively infiltrate throughout the tumor and reach distal cells (Figure 5(a) (3)). Extracellular-dependent transport of conjugates has been further described as ‘infection’ between different batches of cells. As shown in Figure 5(b), the Cy5 signal from PBEAGA^Cy5^-CPT was high in cells on coverslips (ii) and (iii), indicating that some of the PBEAGA^Cy5^-CPT absorbed in cells onto coverslip (i) was secreted into the medium, subsequently becoming endogenous in cells on coverslips (ii) and (iii).

## 3. Strategies for promoting penetration of nano-drugs

A large number of cancer nano-drugs have entered clinical application, including nanoparticulate albumin-bound paclitaxel (Abraxane) (Von Hoff et al., 2011), PEGylated liposomal doxorubicin (Doxil) (Cohen et al., 2012), and liposomal daunorubicin (DaunoXome) (Forssen, 1997). These nano-drugs accumulate at the sites of tumors via the enhanced permeability and retention (EPR) effect (Brigger et al., 2002), but a technique that ensures cancer cells ingest anti-cancer nano-drugs effectively remains an important problem currently restricting the efficacy of cancer treatments. Therefore, the focus of nano-drug design should be methods that increase drug uptake by cancer cells, and enhancement of penetration of the drugs into tumors. To improve the deep penetration of drugs, many studies have focused on drug carriers. Research has demonstrated that the remodeling of the tumor microenvironment (Goetz et al., 2011), charge inversion (Xiao et al., 2011), dimensional change, and surface modifications (Ma et al., 2012), among other methods, can improve one or more of the steps in the CAPIR cascade. Combining these methods can greatly improve the therapeutic effect of drugs on cancer, and also provides a practical reference for cancer treatment.

### 3.1. Remodeling the microenvironment of tumor tissue

The cellular microenvironment consists of the intercellular substances of a cell, the cytoplasm of the cell itself, and the external environment of other cells around it (Allavena et al., 2008). Tumors and their environments are both interdependent and mutually reinforcing, while also being antagonistic and resisting each other. This is of great importance not only for understanding the occurrence, development, and metastasis of tumors, but also for their diagnosis, prevention, and prognosis of patients. Compared with normal tissues, tumor tissue has an anomalous vasculature, rigid extracellular matrix, exhibit hypoxia, a weakly acid pH, and immunosuppressive conditions (Overchuk & Zheng, 2018; Zhou et al., 2020). Microenvironment of tumor tissue plays an important role in the entry of nano-drugs into tumor cells. The concentration of tumor-associated fibroblasts (TAF) in tumor microenvironments is very high. They secrete extracellular matrix consisting of collagen, laminin, and fibrin (Xing et al., 2010). Nano-drugs must pass through this ‘barrier’ to enter the tumor cells. In addition, the microenvironment also contains fibroblasts (Kalluri & Zeisberg, 2006), immune cells (Galon et al., 2006), vascular endothelial cells (Palmer et al., 1988), stellate cells, and other cells, which affect the efficacy of nano-drugs. Therefore, researchers utilize the characteristics of tumor tissue microenvironment to design carriers with the aim of improving the anti-tumor effect.

**Figure 6.**
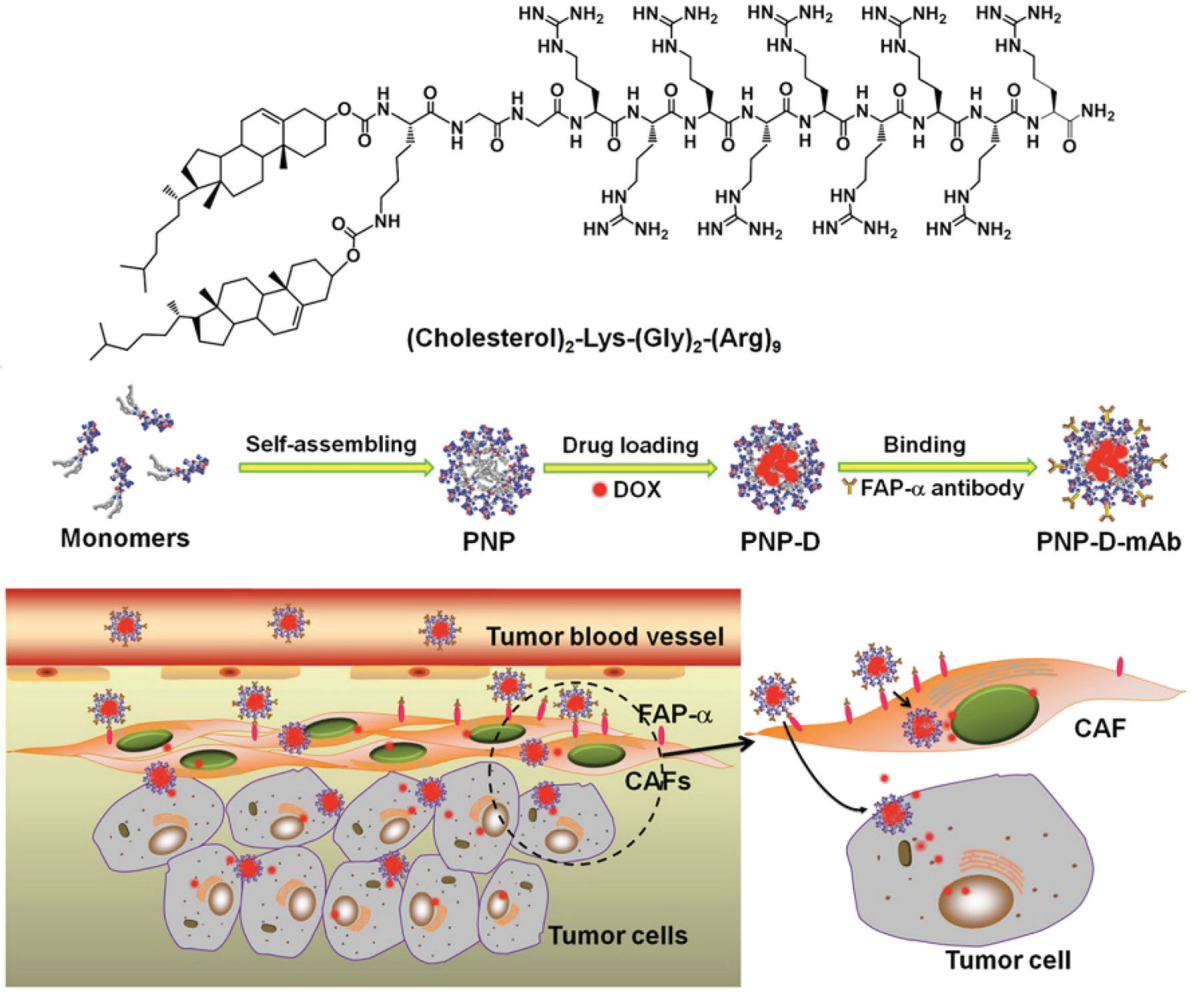
A dual-mode nano-drug with the ability to target CAFs with efficienttumor penetration (Ji et al., 2015).

**Figure 7.**
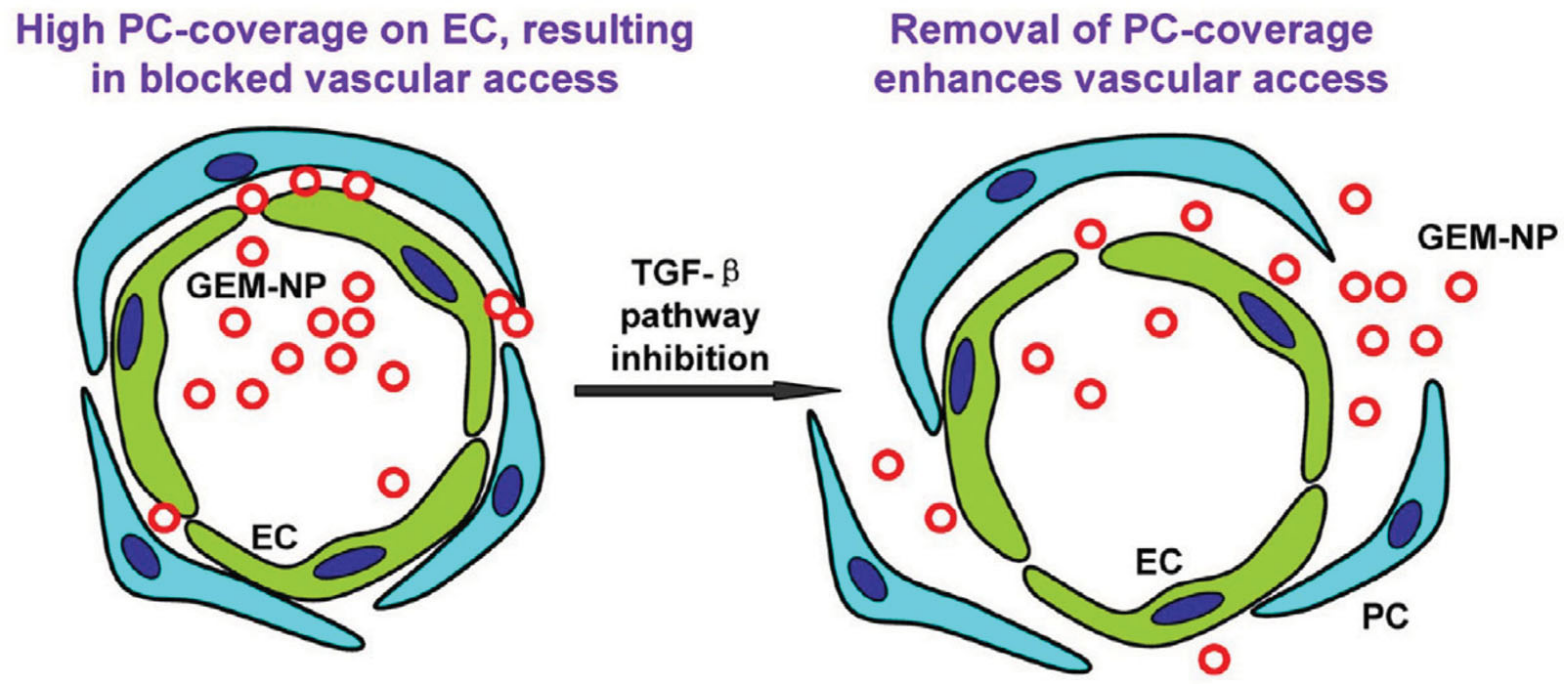
Role of the TGF-β signaling pathway and the effects of pathway inhibition on drug penetration (Meng et al., 2013).

The dense extracellular matrix is the first barrier that nanomedicines face when leaving blood vessels within tumors. The density of tumoral stroma is considerably greater than that of normal tissue, and so nano-drugs remain on the surface of the tumor and do not diffuse deeply within its tissues. Diop-Frimpong et al. (2011) found that after the in situ injection of the collagen inhibitor-losartan into tumors, matrix density within the tumor tissue declined significantly, and diffusion of the nano-drug DOXIL into the tumor was greatly enhanced. Ji et al. (2015) introduced a FAP-α antibody onto the surface of nano-drugs to target tumor angiogenesis factor (TAF). In addition, with the help of cell-penetrating peptide R8, the nano-drugs were rapidly internalized by the TAFs. The nano-drugs had been designed to deactivate the TAFs and successfully improve the permeability of the tumors to nano-drugs resulting in improved anti-tumor efficacy (Figure 6). The tumor microenvironment in pancreatic cancer is rich in connective tissue, accounting for approximately 80% of the total (Bennewith et al., 2009). This dense tumor interstitium not only results in the tumor cells developing resistance to a variety of drugs but also severely limits the penetration of nano-drugs into the tumor tissue (Zhou et al., 2017b). Meng et al. (2013) found that prior to nano-drug treatment, pre-injection of the TGF-β signal inhibitor, LY364947, was able to reform the pancreatic tissue microenvironment, reducing the density of the tumor stroma and reducing coverage of blood vessels by adventitial fibroblasts in the tumor microenvironment, promoting penetration of nano-drug into tumor tissues and enhancing the curative effect of nano-drug treatment (Figure 7).

In the tumor tissue, excessive secretion of tumor vascular endothelial growth factor (VEGF) promotes the formation of a large number of new blood vessels, which generally have wider vessel wall than that of normal blood vessels. There are two opposing strategies for tumor treatment: vascular disruption and vascular normalization. Vascular disruption can be achieved by injecting vascular disrupting agents into the tumoral stroma, such as 5,6-dimethylxanthenone-4-acetic acid (DMXAA) and combretastatin A4 phosphate (CA4P), etc., resulting in insufficient blood supply to the tumor which subsequently kills it. For vascular normalization, anti-angiogenic drugs such as Cediranib, Sunitinib, Bevacizumab, and DC101 can be utilized to reduce vascular penetration and concentration of the matrix, which is conducive to the penetration of nano-drugs allowing treatment of the tumors.

Studies also have shown that hypoxia hinders the formation and metastasis of blood vessels, increasing drug resistance, and leading to the failure of radiotherapy and photothermal therapy. There are several ways to reduce hypoxia, including improving blood flow, delivering oxygen, in situ oxygen production and reduction of oxygen consumption, etc. Photothermal therapy (PTT) can increase blood flow and relieve hypoxia, while delivering oxygen carriers to overcome the resistance of hypoxia-mediated tumor radiotherapy. In addition, oxygen production in situ at the site of a tumor can be achieved through use of MnO2, CaO2, catalase, or photo-driven water lysis. A weakly acidic pH is also a defining characteristic of tumors, and which also promotes their spread and migration. Studies have demonstrated that the aggressiveness of a tumor can be reduced through use of sodium bicarbonate, imidazole, and lysine as acidity buffers to adjust their pH.

In addition, tumor cells can secrete a variety of immunosuppressive factors, inhibit the secretion of regulatory cytokines, and regulate the activity of immune effector cells. This will protect tumor cells from specific cytotoxic T lymphocytes (CTL), thereby promoting tumor growth. For example, transforming growth factor-β (TGF-β), interleukin 10 (IL10), and vascular epidermal growth factor (VEGF) (Phuengkham et al., 2019) secreted by tumor associated macrophages (TAMs), regulatory cells (Tregs) and myeloid-derived suppressor cells (MDSCs) which have the effect of negatively regulating the body’s immune response to tumors and promoting tumor growth. Reshaping tumor immune microenvironment provides a new idea for cancer treatment. The research showed that low-dose gemcitabine can selectively eliminate MDSCs in mice bearing mesothelioma without killing T cells and B cells. On the one hand, gemcitabine can kill tumor cells and reduce tumor burden, on the other hand, it can eliminate MDSC in the body and effectively relieve immunosuppression. In addition, indoleamine 2,3-dioxygenase (IDO), matrix metallo-protease (MMP), TGF-β and chemokines CXCL12 and other soluble mediator-related inhibitors have also received extensive attention in regulating tumor immune microenvironment (Zhou et al., 2020).

### 3.2. Charge inversion

The surface of a cell is highly negatively-charged, and so researchers have hypothesized that a positively-charged drug carrier would promote endocytosis of the drug by cancer cells due to the principle of mutual attraction between heterogeneous charges. However, this approach leads to the recognition by the reticuloendothelial system in the blood circulation, accelerating its rate of clearance (Chen et al., 2017). Several groups have studied the effects of surface charge on the pharmacokinetics polymer nanoparticles and their total accumulation in tumors, finding that inherently neutral or negatively charged nanoparticles were more likely to accumulate in tumors. In addition, negatively charged or neutral nanoparticles were found to circulate for longer in blood. Therefore, dynamic charge strategies should be designed to regulate the efficacy of nano-drugs. By comparing the penetration of PEGylated nanoparticles with different charges in tumor tissues, Miura et al. (2014) found that weak positive charges on the surface of nanoparticles greatly increased their capability to penetrate tumor tissues. Positive charges on nano-drugs improved their capability to penetrate into cells and achieve intracellular drug release, an effective method of solving the three-step PIR process described above for drug delivery. However, positively charged nanoparticles in the blood are quickly cleared by macrophages and the mononuclear phagocytic system (MPS), resulting in an overly short duration in the blood circulation. Therefore, positive charges of the carrier should be shielded in the blood circulation system by neutral or negative charges in order to accomplish ‘invisibility.’ Such a charge reversal strategy is currently the principal method used to solve this problem.

Tumor tissues create an acidic microenvironment, the pH of tumor stroma being between 6.5 and 7.4. Researchers have attempted to modify the surface of nano-drug carriers with an acid-sensitive anhydride. In the neutral environment of blood, the surface of nano-drugs is negatively charged. When accumulating in tumor tissue due to the EPR effect, the acid-sensitive anhydride is shed, exposing amino groups that are positively charged, promoting endocytosis and tumor penetration. Using acid-sensitive 2,3-dimethyl maleic anhydride (DMMA) on the surface of nano-hydrogels, Du et al. (2010) fabricated hydrogels that were negatively charged in a neutral environment. Within tumor tissues, the hydrogels were hydrolyzed to become positive, improving endocytosis of the nano-drugs and promoting their penetration into tumor cells. Koren et al. (2012) prepared a multifunctional target transmembrane liposome system with a nucleosome-specific monoclonal antibody conjugated to PEG3400-phospholipid and cell-penetrating peptide (TATp). The hydrazone bond in system remained stable in the blood circulation (pH 7.4), the TATp fragment effectively shielded by the long-chained PEG3400. In the extracellular acidic environment of the tumor, the hydrazone bond was hydrolyzed, removing the long chain of PEG3400, exposing TATp which effectively mediated penetration of liposomes into target cells and greatly enhancing penetration capability of the nano-drug into tumor cells, further promoting the endocytic process. A variety of other pH-sensitive groups and polymers is presented in Table 1.

**Table 1.** pH-responsive chemical bonds and groups (Liu et al., 2016).

	pH = 6.5–7.2	pH = 4.5–6.5
pH-sensitive group	Polysulfonamide Polyhistidine	Maleic acid derivative Acrylic derivative
PH-sensitive chemical bonds		

**Figure 8.**
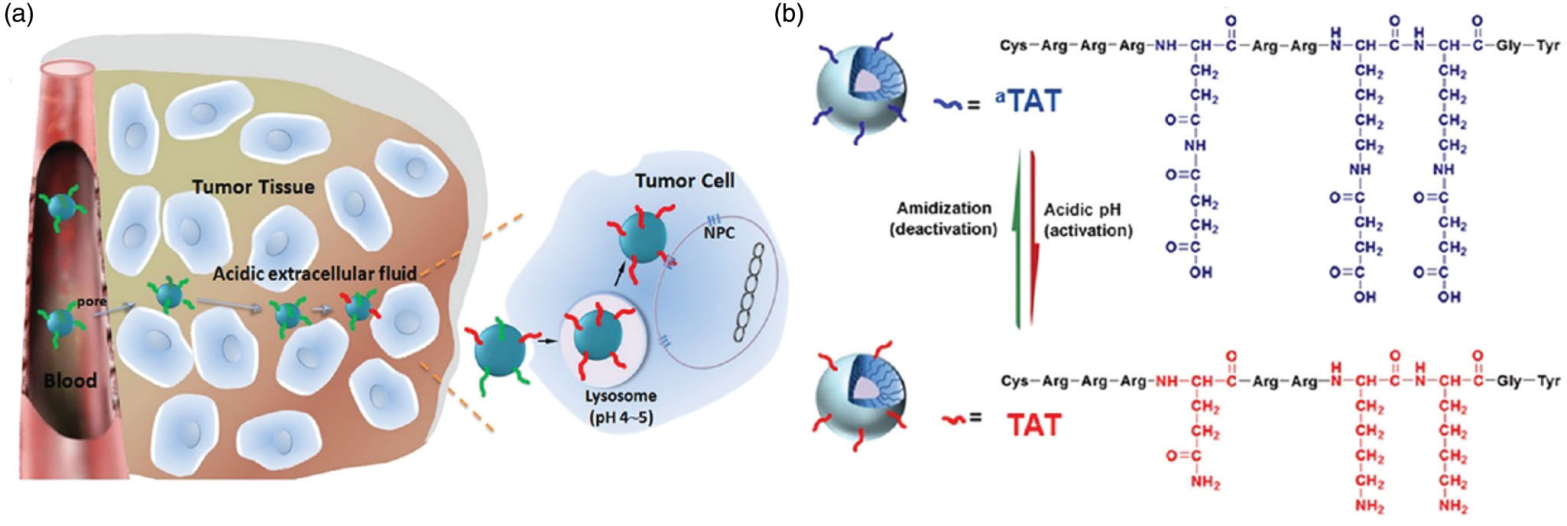
(a) Illustration of the use of TAT as an example of a cell-penetrating peptide (CPP) to demonstrate the concept of CPP deactivation in the blood compartment and its activation in the tumor interstitium or cells to acheive in vivo tumor-targeted drug delivery. (b) Amidization of TAT’s primary amines to succinyl amides for acid-triggered hydrolysis (Jin et al., 2013).

Jin et al. (2013) designed a drug carrier that greatly increased cell penetration. They first modified the TAT cell-penetrating peptide which contains a large proportion of lysine, the amine of which is amidated, not only achieving a stealth effect by the nanocarrier in the blood circulation but also inhibiting nonspecific interactions. The modified cell-penetrating peptide, referred to as ^a^TAT, retains strong cell-penetrating capability. In addition, ^a^TAT was attached to PEG-PCL for the preparation of micelles in which the nano-drugs were encapsulated (Figure 8). In this way, an uncharged drug-delivery system for application in the blood circulation was constructed. Using the EPR effect, the drug-loaded system was able to accumulate close to the tumor. Because the tumor microenvironment is weakly acidic, the amides were hydrolyzed, regenerating the original function of the cell-penetrating peptides in the extracellular fluid of the acidic tumors, exposing amino groups, causing the entire system to become positively charged. In this way, it bound the negative charges on the surface of the cancer cells, thereby greatly enhancing endocytosis of the drugs, increasing their accumulation in tumor cells, and ultimately improving treatment efficacy.

However, the acidic microenvironment in tumors, with a pH of less than 7.0, is located far from the tumor capillary network and undergoes hypoxia, while the pH close to the capillaries is close to normal (almost 7.4). As a result, nanomaterials ooze from tumor capillaries and remain within the normal pH of the interstitium, unable to undergo charge reversal and unable to diffuse into the acidic regions, causing them to accumulate close to the tumor blood vessels. Therefore, nanomaterials with high tumor permeability must also be able to exude from the tumor capillaries and generate positive charge reversal. Therefore, identification of a ‘signal’ close to tumor capillaries capable of triggering charge reversal is key to anti-tumor efficacy. There are sufficient nutrients and oxygen surrounding tumor capillaries, so that the tumor cells can be highly metabolically-active with rapid proliferation, causing them to overexpress a variety of specific enzymes, such as primordium glutamyl transpeptidase, matrix metalloproteinase, and aminopeptidase N, *etc* (Choi et al., 2012). Therefore, enzyme-catalyzed amine groups attached to the nano-drugs ensure positive charges on the nano-drug following enzyme catalysis. Enzyme-responsive charge-reversal elements must be electrically neutral to ensure long-term persistence in the blood circulation (Dong et al., 2019).

**Figure 9.**
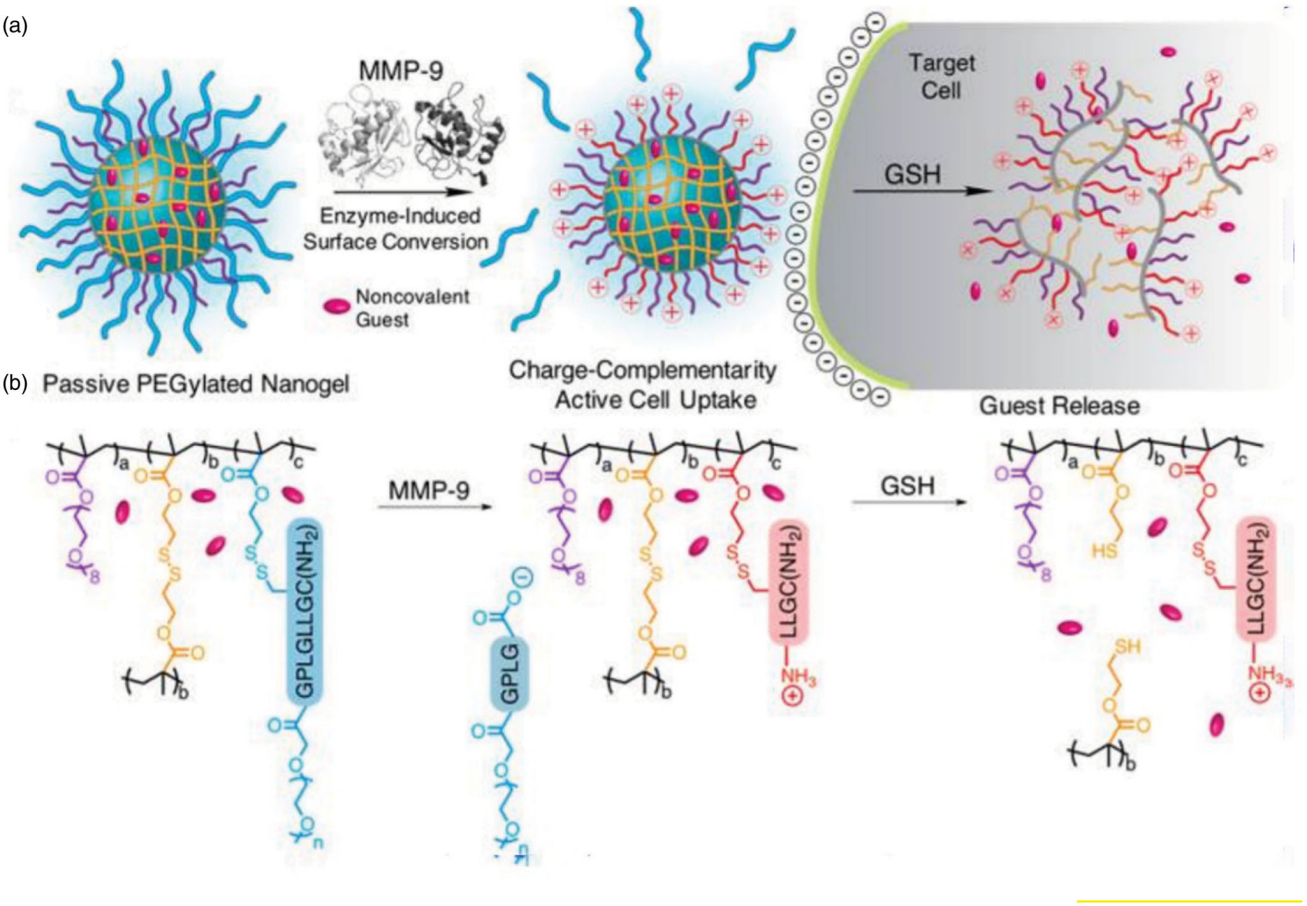
MMP-9 response charge reversal gel system showing a more rapid rate of endocytosis rate and representing a passive PEGylated gel system (Gordon et al., 2018).

Zhou et al. (2019) designed a series of GGT-responsive vectors. Firstly, a GGT-responsive molecule and CPT nano-drug were attached to PEG-PCL, using the self-assembly of block copolymers to form micelles, and preparing two drug-loading systems, PBEAGA-CPT and PEAGA-CPT. Being negatively charged, these two systems exhibited a greatly prolonged duration in the blood circulation. Within a specific tumor site that has high levels of GGT, the γ-glutamyl in PBEAGA-CPT is immediately hydrolyzed to release hidden amino groups, causing the entire drug-loaded system to become positive. Electrostatic interactions greatly promote endocytosis by the tumor cells, resulting in substantial accumulation in the cells, enhancing therapeutic efficacy. However, when placing the PEAGA-CPT drug-loaded system in an environment with high GGT expression, charge reversal did not occur and in subsequent animal experiments, a therapeutic effect inferior to that of PBEAGA-CPT was observed. Gordon et al. (2018) proposed a simplified protease activation strategy to explore the generation of peptide N-termini on the surface of particles, for the promotion of cellular uptake (Figure 9). This can be achieved by attaching a protease-cleavable peptide to a nanogel at its C- and N-termini which is shielded by PEG. The peptide could be hydrolyzed by MMP-9, in which PEG is removed, exposing a polyamine-type surface at the N-terminus. Due to charge conversion, reduced steric stabilization, and enhanced membrane interaction, the synthesized ‘active’ nanogels can be expected to internalize faster than ‘passive’ pegylated nanogels.

### 3.3. Dimensional change

Rational adjustment of the physical and chemical properties of nanoparticles, and modifying their size and shape provides an alternative solution to the problem of penetration (Chauhan et al., 2011; Lee et al., 2013; Blanco et al., 2015). A large number of studies have established that large nanoparticles generally exhibit the EPR effect and have a higher tendency to diffuse, accumulating close to the blood vessels of tumors, but with poor penetrability and dispersibility in dense tumor stroma (Johnston et al., 2003; Perrault et al., 2009; Wang et al., 2015). The size of nanoparticles has a substantial effect on endocytosis in cancer cells. An appropriate size helps promote the accumulation of drugs in the tumor, inappropriate size being counterproductive (Zutter et al., 1998; Choi et al., 2006; Wong et al., 2011). Studies have shown that the particle sizes of polymer micelles after drug loading is larger than those that are unloaded, so that the entire drug loading system requires careful consideration (Shuai et al., 2004a, 2004b; Elhasi et al., 2007). Nano-drugs range in diameter from a few nanometers to more than 100 nanometers. Because diffusion rate is inversely proportional to diameter, the diffusion of large nano-drugs is considerably smaller than that of small molecules (Ceradini et al., 2004). In particular, drugs smaller than 20 nm have reduced resistance to diffusion and therefore have better penetration capability within tumor tissues. In addition, they are taken up more rapidly than larger particles (Chithrani & Chan, 2007; Jiang et al., 2008; Jin et al., 2009). However, such small nano-drugs also have a number of drawbacks, such as a short duration within blood circulation due to their more rapid removal from the blood circulation. Large (>100 nm) nanoparticles can exist for long periods in the blood circulation, but their cell penetration capability is very poor, which are contradictory effects. Therefore, a solution would be the design of a drug carrier with a large particle size during blood circulation which quickly reduces in size in the vicinity of tumor cells (Cabral et al., 2011).

**Figure 10.**
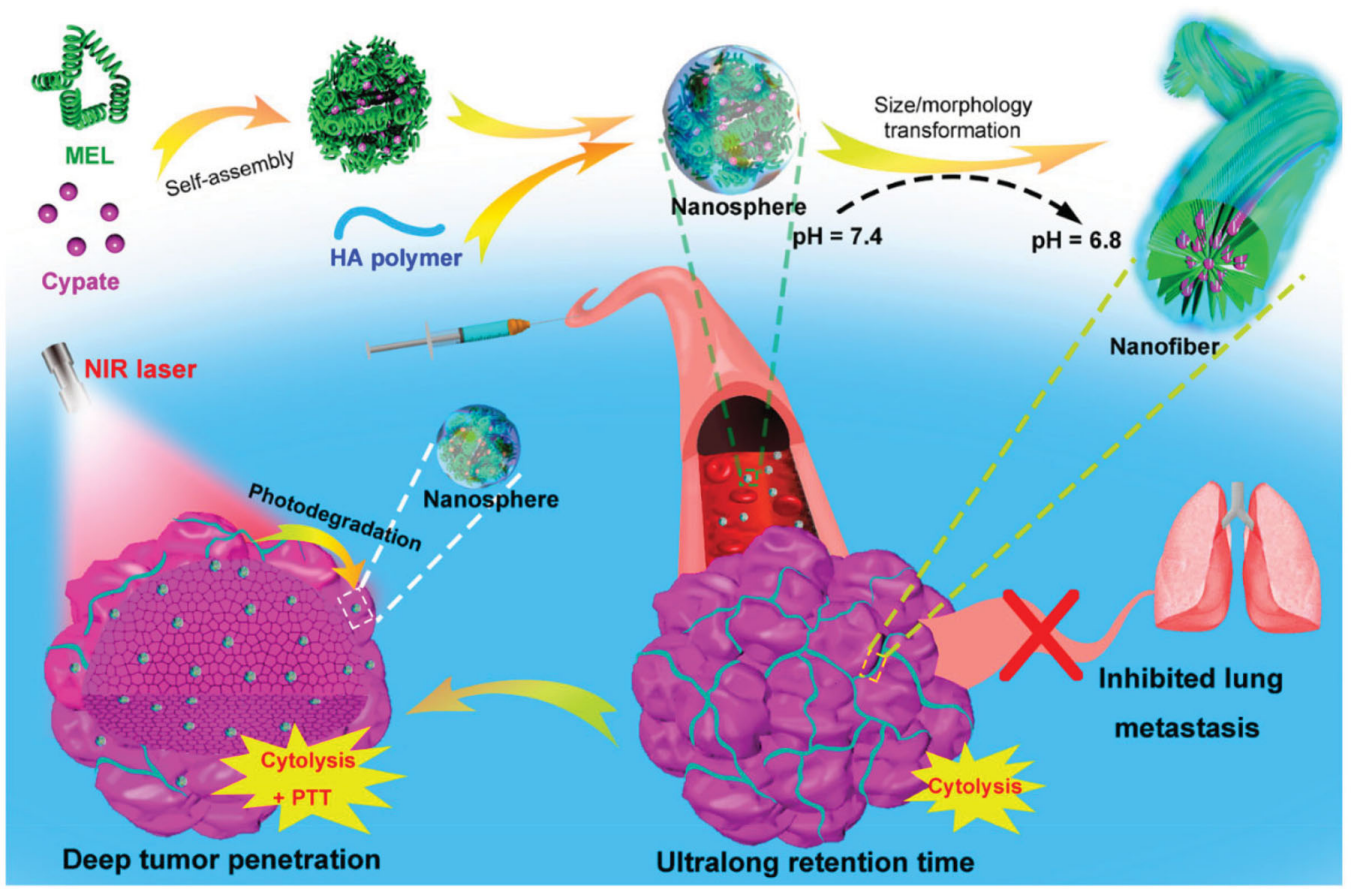
Schematic illustration of the preparation of MEL/Cypate @ HA complex and continuous size/morphology transition induced by weakly acidic tumor microenvironments and near-infrared laser irradiation (Jia et al., 2019).

**Figure 11.**
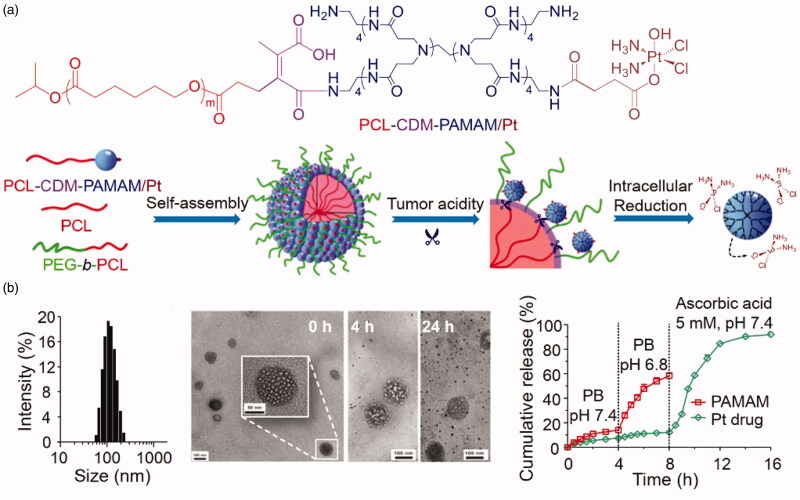
(a) Self-assembly and structural change of PCL-CDM-PAMAM/Pt in response to tumor acidity and an intracellular reductive environment. (b) Self-assembly and structural change of iCluster/Pt in response to tumor acidity and an intracellular reductive environment (Li et al., 2016a).

Wu’s group developed a dual-response nanocarrier with variable size and structure. Nanocarriers were formed by self-assembly of the cell-lysing peptide melittin, the near-infrared photothermal molecule cypate, and hyaluronic acid (HA) polymers with tumor-targeting capability (Figure 10) (Jia et al., 2019). At a pH of 7.4, nanospheres with a particle size of approximately 50 nm are formed, helping to achieve a long duration of circulation of the nano-drugs in blood. When specifically targeted to a tumor site, the particles transform *in situ* from nanospheres to nanofibers due to the slightly acidic environment of the tumor (pH = 6.8), greatly promoting endocytosis in the cells, causing the accumulation of nano-drugs in tumor cells and improving treatment efficacy. Similarly, Li et al. (2016a) designed and synthesized a clustered nano pharmaceutical system, PCL-CDM-PAMAM(iCluster)/Pt, with ultra-sensitive responsiveness to tumor microenvironments (Figure 11). They attached a few nanometers of PAMAM dendrimer to polycaprolactone via an acid-responsive chemical bond through a series of chemical modifications (Cong et al., 2019), then further co-assembled it with PEG-PCL and PCL to obtain a clustered nanocarrier, iCluster/Pt. Within the blood circulation, the size of the nanocarrier was maintained at approximately 80 nm, effectively avoiding recognition by the reticuloendothelial system. Therefore, it was designed to persist within the blood circulation for long periods. Finally, the drug became enriched within the tumor after arriving within its vicinity. Since the pH was acidic, the diameter of the carrier reduced to approximately 10 nm, allowing more efficient uptake of the drug into the cancer cells.

**Figure 12.**
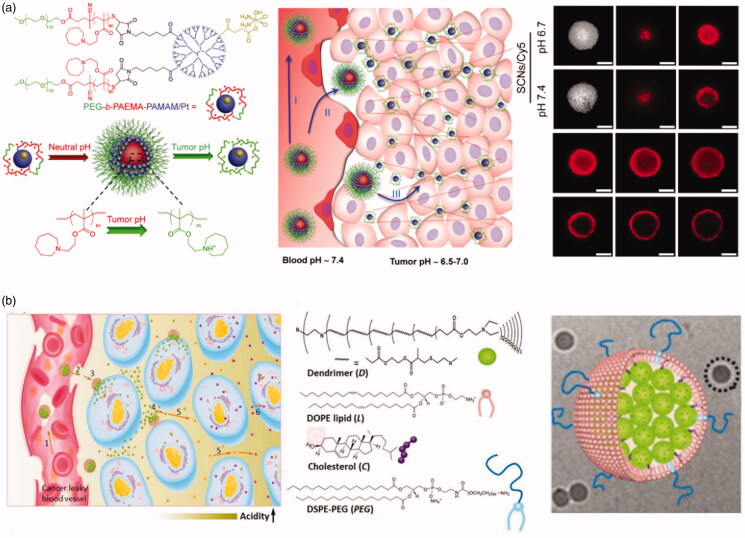
(a) Schematic illustration showing pH-sensitive cluster nano-bombs (EG-b-PAEMA-PAMAM/Pt), a robust nano-platform to overcome biological barriers to drug delivery in poorly permeable tumors, and CLSM images of fluorescence-labeled PEG-b-PAEMA-PAMAM/Pt/Cy5 (Li et al., 2016b). (b) Schematic diagram of the cluster-bomb-like nanoassembly and how it accomplishes the CAPIR cascade. Nanoassembly structure: the dendrimers were self-assembled with DOPE and DSPE-PEG lipids in addition to cholesterol to form a nanoassembly with a dendrimer core and lipidic shell, as confirmed by cryo-TEM imaging (Sun et al., 2014).

Researchers have also found that the optimum size of a nano-drug for endocytosis depends strongly on the cell line under investigation. For example, the uptake of PLGA-DNA complexes by Caco-2 cells depends on their size. Uptake was found to be greatest with particles with a mean diameter of 100 nm (Desai et al., 1997). However, uptake was highest in COS-7 and HEK293 cell lines for particles with mean diameters of 70 nm and 200 nm, respectively. Therefore, it is necessary to consider the cell type the carrier is targeting (Prabha et al., 2002). Li et al. (2016b) synthesized the pH-responsive nanocarrier PEG-b-PAEMA-PAMAM/Pt (Figure 12(a)), in which the PAEMA block was pH-sensitive. In the neutral environment of the blood circulation, the PAEMA block was hydrophobic, forming nano-micelles by directed self-assembly of PEG-b-PAEMA-PAMAM/Pt into SCNs/Pt, with a mean particle diameter of approximately 80 nm, allowing extended duration within the blood circulation (Zahr & Pishko, 2007; Pei et al., 2010). Upon reaching the tumor, PAEMA rapidly protonates and becomes hydrophilic in the weakly acidic environment (Lee et al., 2008), causing SCNs/Pt to instantly disintegrate into small nanoparticles that can penetrate the tumor. CLSM images displaying in vitro penetration of fluorescence-labeled PEG-b-PAEMA-PAMAM/Pt/Cy5 into tumor cells are shown in Figure 12(a), which demonstrates deep penetration and uniform distribution of Cy5 at pH 6.7 following rapid disintegration into small particles.

Shen et al. designed and fabricated a ‘bullet-shaped’ liposome assembly with small dendrimers that fill ‘bullet’-like liposomes (Figure 12(b)). This approach was effective in avoiding the drawback of the rapid clearance of small nanoparticles from the blood, allowing the liposomes to process of drug delivery can be efficient, finally demonstrating excellent anti-tumor activity in *in vivo* tumor inhibition experiments and a long time circulatation. After reaching and penetration of nano-drugs in the tumor tissue. After reaching and aggregating at the tumor due to the EPR effect, dendrimers of several nanometers within liposomes were released, promoting the penetration of nano-drugs in the tumor tissue. Thus, the CAPIR process of drug delivery can be efficient, finally demonstrating excellent anti-tumor activity in in vivo tumor inhibition experiments. In addition, we also summarized the strategies for constructing dimensional changed drug delivery systems, as shown in Table 2.

**Table 2.** Strategies to construct dimensional changed drug delivery systems (Yu et al., 2020).

Methods and strategies	Responsive molecule
Aggregation strategies	
Enzyme	Matrix metalloproteinase, legumain, hyaluronidase (HAase), gelatinase, furin, and caspase 3/7
pH	Hydrolysis-susceptible citraconic amide, 11-mercaptoundecanoic acid, and (10-mercaptodecyl)-trimethylammonium bromide)
Light	Azobenzene, spirobenzopyran, triphenylmethane, and cinnamenyl
Temperature	PPCs, PNIPAm, PDEAm, PEO, PPO, and polyphosphoesters
Redox	Disulfide bond with GSH
Size-shrinkage strategies	
pH	Amino polymers, DMA, and Schiff base
Enzyme	MMPs, HAase, amylase, and thrombin
Redox	disulfide bond
ROS	Thioketal, thioester, polypropylene sulfide, and phenylboronic ester

### 3.4. Surface modification

**Figure 13.**
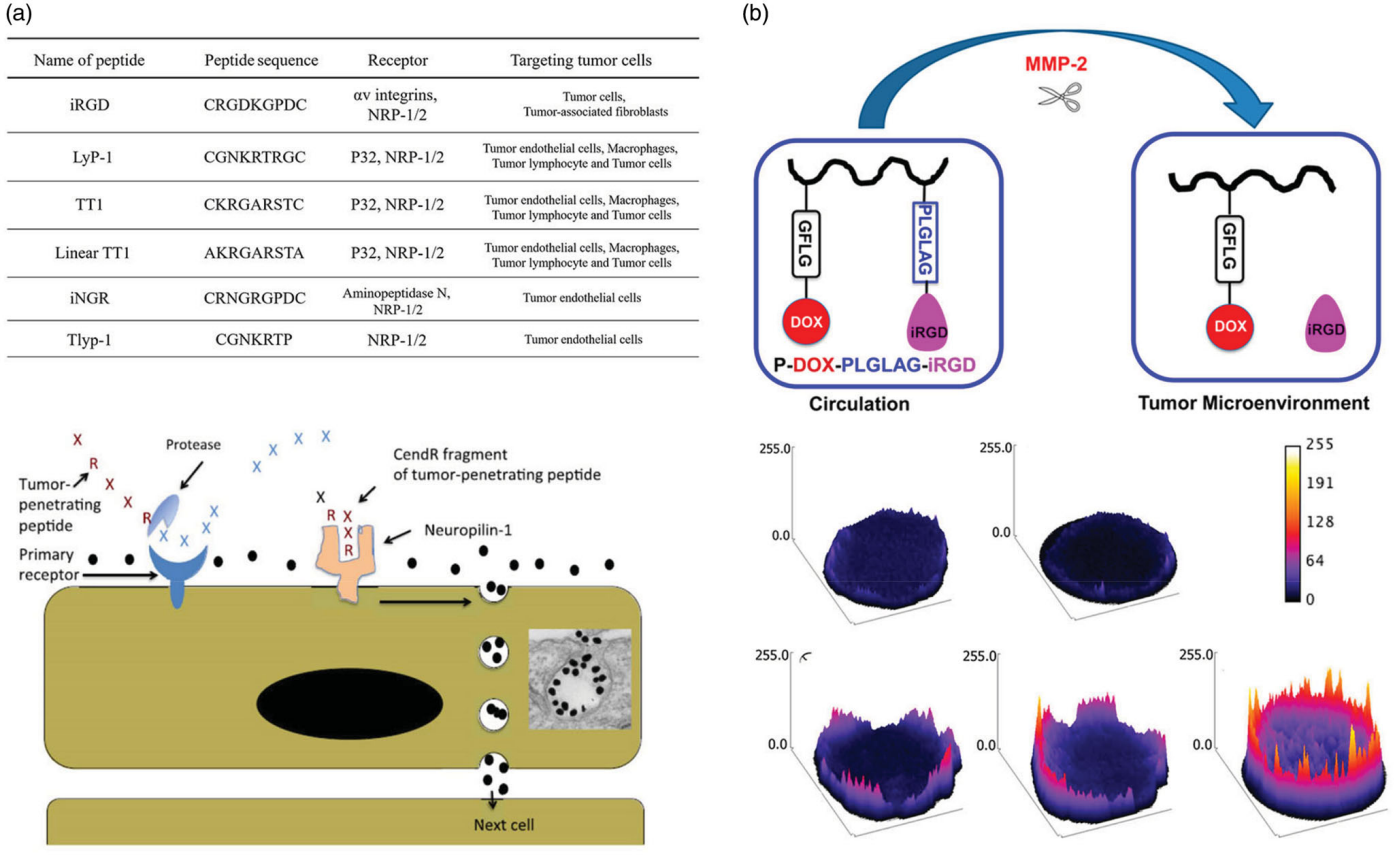
(a) Peptides containing the sequence (R/K)XX(R/K) are effective at penetrating tumor tissues (Ruoslahti, 2017). And schematic representation of the CendR trans-tissue transport pathway(Qiao et al., 2018). (b) Schematic illustration and proposed fate of P-DOX-PLGLAGiRGD, and penetration of DOX conjugates and controls in DU-145 MTS, control, iRGD, P-DOX, PDOX + iRGD, and P-DOX-PLGLAG-iRGD (Peng & Kopecek, 2015).

Targeted groups of nanoparticles can specifically identify tumor cells, trigger receptor-mediated endocytosis, accelerate the distribution of nano-drugs in tumor tissues, and promote their deep penetration (Liu et al., 2015; Ruoslahti, 2017; Zhang et al., 2020). Many peptides that are effective for penetration in tumor tissues contain the sequence: (R/K)XX(R/K) (Figure 13(a)(1)), where X represents an amino acid other than lysine or arginine. These peptides are also known as CendR peptides. As shown in Figure 13(a)(2), the principle of rapid infiltration of CendR peptides into tumor tissues is as follows: (i) the peptide binds to the primary receptor on the surface of tumor endothelial cells. For example, the primary receptor of iRGD is an αvβ3/αvβ5 integrin, and that of the Lyp-1 peptide sequence is p32/gC1qR. (ii) the CendR sequence of the polypeptide becomes exposed by hydrolysis of the protease (C-terminal); (iii) the CendR sequence binds to neuropilin-1 (NRP-1) on the cell surface, which becomes rapidly integrated into the cell to be subsequently excreted out by exosmosis, where it ‘infects’ other adjacent tumor cells for rapid infiltration into the tumor tissue.

Based on the targeted binding and internalization mechanism of CendR described above, exogenous nanomaterials can be transported effectively to tumor tissues by corresponding peptides on surface-modified nanomaterials. Peng & Kopecek (2015) prepared matrix metalloproteinase 2 (MMP-2)-responsive N-(2-hydroxypropyl)- methacrylamide (HPMA) copolymer drugs and tumor penetrating peptide conjugates (P-DOX-PLGLAG-iRGD). Significant improvement in the permeability of HPMA to tumor tissues was achieved by partial modification of the surface of HPMA with iRGD peptides (Figure 13(b)). Similarly, Li *et al.* attached the iRGD peptide onto the surface of cell membranes to significantly improve the permeability of red blood cell-based drug nanoparticles in tumor tissues and improve the *in vivo* therapeutic effect of the nano-drug system (Su et al., 2016). Tumor osmotic peptides, such as tlyp-1 (Liang et al., 2015) and PFVULI (Cai et al., 2014) have also been shown to significantly improve the penetration of nano-drugs into tumor tissues. Gao et al. developed a method for the treatment of breast cancer in combination with the tumor homing peptide iRGD and IDDHN, a type of multistage-responsive penetrating nanoparticle. IDDHN was composed of a hyaluronic acid (HN) shell modified by an NO donor and small-sized dendrimer. iRGD was specific for the target receptor, αvβ3, located in breast cancer cells. The IDDHN/iRGD nanocarrier was variable in size with a particle diameter of approximately 330 nm in the blood circulation but only 35-60 nm within the tumor tissue. They confirmed that its combined use with iRGD significantly improved tumor targeting and penetration capability of IDDHN, achieving satisfactory treatment of tumors.

## Conclusions

The inability of nano-drugs to penetrate deep into tumor tissues as part of the ‘CAPIR’ process remains a bottleneck restricting the development of nano-drugs. This review summarizes recent developments in the penetration of nanomaterials into tumor tissues, presenting regulatory strategies that can provide a solid source of information for the scientific community. Nano-drugs can be transported into tumor tissue by paracellular or transcellular transport and the size of the nano-drugs and pathological features of the tumor tissue remain the principal factors controlling endocytosis. In addition, high recognition of the reticuloendothelial system in the blood circulation will accelerate the rate of clearance and reduce the accumulation in tumor tissue. Hence, to construct a drug delivery system that promotes endocytosis, the core principle is construction of a dynamic strategy (i.e. remodeling the microenvironment of the tumor tissue, charge inversion, dimensional change, or surface modification of ligands). These dynamic strategies can be activated within specific tumor microenvironments (pH, reducing substances, and enzymes). We have summarized the construction methodologies of these dynamic drug delivery systems that promote deep penetration into tumor tissues. It has been shown that drug delivery systems that promote endocytosis achieve the accumulation of nano-drugs in tumor cells and improve cancer treatment efficacy.

Although we can use the special tumor microenvironment to build drug delivery systems, the inevitable problem remains that many nanoparticles in the blood circulation will be uptake into different organs (especially the kidney). Hence, the biocompatibility and safety of a drug system remain the top priority for cancer treatment. The multiple specific conditions in the tumor tissue microenvironment need to be fully exploited to create a more accurately targeted and efficient drug delivery system so that a greater quantity of the drug reaches the tumor and exert their efficacy. Multi-response drug carriers utilize a synergistic effect of different environment-responsive molecules, overcoming various physiological obstacles encountered in drug delivery systems. However, the design, synthesis, and quality control of multi-stimulus response drug carriers are complex. With the development of polymer materials, tumor pharmacology, molecular biology, and other disciplines, these problems will be solved, and intelligent nano-drug carriers will develop toward more accurate drug delivery. The development of penetration strategies for nanomaterials is without doubt far from complete, as many difficulties and challenges in cancer treatment remain. More bioresponsive drug delivery strategies with few side effects and high treatment effect are required.
